# Interleukin-1 receptor associated kinase 1 (IRAK1) is epigenetically activated in luminal epithelial cells in prostate cancer

**DOI:** 10.3389/fonc.2022.991368

**Published:** 2022-09-26

**Authors:** Undraga Schagdarsurengin, Vanessa Breiding, Maria Loose, Florian Wagenlehner, Temuujin Dansranjav

**Affiliations:** ^1^ Clinic of Urology, Pediatric Urology and Andrology, Justus-Liebig-University Giessen, Giessen, Germany; ^2^ Working group Epigenetics of the Urogenital System, Clinic of Urology, Pediatric Urology and Andrology, Justus-Liebig-University Giessen, Giessen, Germany; ^3^ Working group Urological Infectiology, Clinic of Urology, Pediatric Urology and Andrology, Justus-Liebig-University Giessen, Giessen, Germany

**Keywords:** prostate cancer, IRAK1, Myddosome complex, epigenetic regulation, DNA methylation

## Abstract

The use of immune adjuvants such as toll-like receptor (TLR) agonists reflects a novel strategy in prostate cancer (PCa) therapy. However, interleukin-1 receptor associated kinase 1 (IRAK1), a central effector of TLR signaling, has been shown to be responsible for resistance to radiation-induced tumor cell death. In order to better understand the function and epigenetic regulation of IRAK1 in PCa, we performed *in vitro* cell culture experiments together with integrative bioinformatic studies using the latest single-cell RNA-sequencing data of human PCa and normal prostate (NOR), and data from The Cancer Genome Atlas. We focused on key effectors of TLR signaling, the Myddosome-complex components IRAK1, IRAK4 and MYD88 (myeloid differentiation primary response 88), and TRAF6 (tumor-necrosis-factor receptor associated factor 6). In PCa, *IRAK1*-mRNA was specifically enriched in luminal epithelial cells, representing 57% of all cells, whereas *IRAK4* and *MYD88* were predominantly expressed in leukocytes, and *TRAF6*, in endothelial cells. Compared to NOR, only *IRAK1* was significantly overexpressed in PCa (Benjamini-Hochberg adjusted p<2x10^-8^), whereas the expression of *IRAK4*, *MYD88*, and *TRAF6* was unchanged in PCa, and *IRAK1*-expression was inversely correlated with a specific differentially methylated region (*IRAK1*-DMR) within a predicted promoter region enriched for H3K27ac (Spearman correlation r<-0.36; Fisher’s test, p<10^-10^). Transcription factors with high binding affinities in *IRAK1*-DMR were significantly enriched for canonical pathways associated with viral infection and carcinogenic transformation in the Kyoto Encyclopedia of Gene and Genomes analysis. DU145 cells, exhibiting hypermethylated *IRAK1*-DMR and low *IRAK1*-expression, reacted with 4-fold increased *IRAK1*-expression upon combined treatment with 5-aza-2-deoxycytidine and trichostatin A, and were unresponsive to infection with the uropathogenic *Escherichia coli* strain UTI89. In contrast, PC3 and LNCaP cells, exhibiting hypomethylated *IRAK1*-DMR and high endogenous *IRAK1*-mRNA levels, responded with strong activation of *IRAK1*-expression to UTI89 infection. In summary, exclusive overexpression of *IRAK1* was observed in luminal epithelial cells in PCa, suggesting it has a role in addition to Myddosome-dependent TLR signaling. Our data show that the endogenous epigenetic status of PCa cells within *IRAK1*-DMR is decisive for *IRAK1* expression and should be considered as a predictive marker when selective IRAK1-targeting therapies are considered.

## Introduction

Prostate cancer (PCa) is the second most frequent malignancy after lung cancer, and the fifth leading cause of cancer-related deaths among men worldwide (1). Chronic inflammation is commonly observed in normal and malignant prostates and is considered to be one of the main driving mechanisms of prostate carcinogenesis (2, 3).

Toll-like receptors (TLRs) are a family of transmembrane proteins that are the main sensors of innate and adaptive immunity. They are responsible for the activation of pro-inflammatory cytokines and chemokines in inflammatory cells within tumors, as has been shown in hepatocellular, head neck, gastric, and breast cancers (4–7). In the prostate, TLR4 and TLR9 are known to be expressed in normal and malignant cells, thereby enabling prostate epithelial cells to act as immune sensors (8–10).

Under certain conditions, inflammation can counter tumor progression, and activation of effective antitumor immunity is considered a potent adjuvant therapy option (10). As recently reviewed, TLR stimulation in combination with chemotherapy or radiotherapy has been shown to be effective against tumor progression in multiple clinical trials (11). Immunotherapy with TLR agonists significantly improves the outcomes of radiation therapy (RT) in preclinical models (11, 12). The antitumor immune response, which is triggered by tumor antigens released from irradiated tumor cells, is considered to be the main mechanism underlying this therapeutic effect (13, 14). The assembly of the Myddosome complex and recruitment of tumor necrosis factor receptor-associated factor 6 (TRAF6) to the Myddosome complex are crucial events that ultimately promote Myddosome-dependent TLR immune signaling (15). The interleukin-1 receptor-associated kinase 1 (IRAK1) is a critical component of the Myddosome complex and is one of the main effectors of TLR signaling. In human cancer cells and a zebrafish model, IRAK1 was shown to be responsible for RT resistance (14). Selective IRAK1 inhibitors, such as the organic JAK2/FLT3 inhibitor pacritinib and chemical compounds, such as Jh-X-119-01, are being considered for cancer treatment (16). However, setting up individualized PCa therapy strategies may be complicated by the molecular heterogeneity of the tumors. Despite its critical role in tumorigenesis and tumor therapy, little is known about PCa-associated epigenetic and transcriptional regulation of genes encoding effectors of TLR signaling. In order to better understand and utilize the TLR-mediated anti-tumor immune response in PCa treatment, it is essential to first characterize the role of epithelial cells in immune signaling and the function of key effectors of TLR signaling.

Here, we analyzed genes encoding key components of the Myddosome complex, including IRAK1, IRAK4, and MYD88, and the TLR adaptor protein TRAF6, using recently published single-cell RNA-seq data of PCa (17), The Cancer Genome Atlas (TCGA) data bank, and *in vitro* cell culture experiments, with a focus on their epigenetic regulation in PCa. We showed that in PCa, IRAK1 plays an exclusive role in the luminal epithelial cell population and is tightly regulated by the epigenetic status of a specific differentially methylated region (*IRAK1*-DMR) within the predicted gene promoter.

## Material and methods

### Analysis of single-cell RNA-sequencing data generated in human PCa

Gene expression matrix files from single-cell RNA sequencing (scRNA-seq) of eight radical prostatectomy (RP) specimens from men with localized PCa were downloaded from the Gene Expression Omnibus (GEO) database GSE176031 (17) (**Table S1**). The data were computationally processed using the Seurat (v4.1) package (18). Cells with fewer than 200 detected genes and mitochondrial levels greater than 5% were excluded from analysis. Doublets were identified using DoubletFinder (v2.03) and were removed. Only the genes expressed in more than three cells were considered. Data were merged using the integration method based on commonly expressed anchor genes using ‘FindIntegrationAnchors’ and ‘IntegrateData’ functions in the Seurat package (19). Subsequent dimensionality reduction, clustering, and visualization were performed using the ‘FindNeighbors’ and ‘FindClusters’ functions of the Seurat package. Cells in clusters were annotated using the AUCell package (v1.16) utilizing cell-type signature gene sets, which were generated by single-cell profiling of the normal prostate (20) (**Table S2**).

Gene expression across the major cell types was visualized using the R package Nebulosa (v1.4) (21), which uses weighted kernel density estimation to recover gene expression signals.

### Analysis of bulk RNA-seq and DNA methylation of human PCa using TCGA

To assess the role of DNA methylation in the expression of *IRAK1*, *IRAK4*, *MYD88* and *TRAF6*, bulk RNA sequencing and 450K Illumina methylation data from 341 PCa and 35 normal prostate tissue specimens from The Cancer Genome Atlas (TCGA) were utilized. Supplemental clinical data, raw methylation data (‘IDAT’) and RNA-seq data (‘HTSeq-counts’) were extracted from TCGA using the R package ‘TCGAbiolinks’ (v2.18.0). Data pre-processing and subsequent analyses were performed using the R packages ‘minfi’ (v1.36.0) and ‘missMethyl’ (v1.28) for 450K Illumina methylation data, and ‘Biobase’ (v2.50) for RNA-seq data. Furthermore, *IRAK1* expression was analyzed in castration-resistant prostate cancer metastases (mCRPC, n=99) (TCGA: Project ID WCDT-MCRPC). CRPC-related overexpressed hub genes, including *TARDBP, HNRNPA2B1, MRPS25, MAPK8IP3, CCDC14* and *GOLGA8B*, which were also found to be associated with PCa progression and prognosis (22), were used to select non-CRPC-like PCa specimens (N=194) within TCGA. Differential expression analysis was performed using the empirical Bayes statistics (eBayes) function in the ‘limma’ package. P-values were adjusted for multiple comparisons using the Benjamini-Hochberg (BH) method. Differentially methylated regions were identified using the DMRcate package (v2.8.4), hidden Markov model and Fisher exact test (HMM-Fisher). For illustration, ChAMP graphical user interface was used (23).

### Cell culture experiments

The human PCa cell lines PC3, LNCaP, and DU145 were obtained from German Collection of Microorganisms and Cell Cultures GmbH (DSMZ, Braunschweig, Germany). DU145 and LNCaP cells were cultured in RPMI 1640 medium (Gibco), and PC3 in DMEM (Gibco) supplemented with 10% fetal bovine serum (Gibco) and 1% penicillin/streptomycin (Gibco). Cells were cultured in 10 cm dishes in 5% CO2 at 37°C to 70–90% confluency and then used for subsequent analyses.

The effects of the DNA methyltransferase 1 (DNMT1) inhibitor 5-aza-2′-deoxycytidine (5-AZA) (Sigma Aldrich Corp, St. Louis, MS, USA) and the histone deacetylase (HDAC) inhibitor trichostatin A (TSA) (Upstate Biotechnology, Lake Placid, NY, USA) on the expression of *IRAK1* were examined in PCa cell lines. Cells were grown to 40% confluency and treated with 5µM 5-AZA for 96 h and/or with 0.3µM TSA for 24 h. Total RNA was extracted from untreated and treated PC3, DU145, and LNCaP cells using peqGOLD TriFast reagent (VWR, Radnor, PA, USA) according to the manufacturer’s protocol. Reverse transcription (RT) was performed on 1 µg of total RNA using M-MLV transcriptase and an adjusted buffer system (Promega, Madison, WI, USA), random hexamers, and poly-dT primers for 1 h at 42°C. Quantitative PCR (qPCR) was performed using 50 ng of cDNA per PCR in a Rotor-Gene Q PCR Cycler (Qiagen, Hilden, Germany) with *IRAK1* and *GAPDH*, as reference genes (**Table S3**). Relative *IRAK1* expression was calculated by normalizing to *GAPDH* using the 2 -ΔΔCt method.

*IRAK1* promoter methylation was analyzed in the PCa cell lines. DNA was isolated from proteinase K-treated cells using a standard phenol-chloroform extraction method. In total, 2µg of DNA was bisulfite-treated using an EZ DNA Methylation Kit (Zymo Research, Irvine, CA, USA). Specific DNA fragments representing *IRAK1*-DMR with six CpG sites were amplified using the appropriate primer sets (**Table S3**). The PCR products were separated on a 2% agarose gel according to product size and pyrosequenced using the PyroMark Q24 System (Qiagen). Methylation values were analyzed using the PyroMark Q24 Software (Qiagen). The degree of methylation for each CpG site was calculated in a program as the percentage of methylated cytosines over the sum of the total cytosines at the indicated single CpG site.

The uropathogenic *Escherichia coli* strain UTI89 was obtained from DSMZ and used to infect PCa cell lines PC3, LNCaP, and DU145. In total, 1×10^6^ PCa cells cultured in 6-well plates were infected with UTI89 at a multiplicity of infection (MOI) of 10 for two and four hours. Untreated and treated cells were washed twice with phosphate-buffered saline and lysed with peqGOLD TriFast reagent (VWR). RNA extraction and RT-qPCR were performed as previously described.

### Analysis of the regulatory function of *IRAK1*-DMR using ENCODE

The regulatory function of *IRAK1*-DMR was analyzed using the Encyclopedia of DNA Elements (ENCODE) and taking into consideration the enrichment of histone 3 acetylated at lysine 27 (H3K27ac) in the predicted *IRAK1* enhancer and promoter region (gathered from Segway and ChromHMM data) and JASPAR-2022 data, a core database of transcription factor binding sites (TF-BSs). Prediction of TF-BSs was made according to the position weight matrix (PWM), and relative scores ≥ 0.8 with a p-value < 0.001 were considered as true TF-BSs. Functional characterization of the identified TFs was performed using the Kyoto Encyclopedia of Genes and Genomes (KEGG).

## Results

### In prostate cancer, *IRAK1* is expressed in luminal epithelial cells

**Figure 1:**
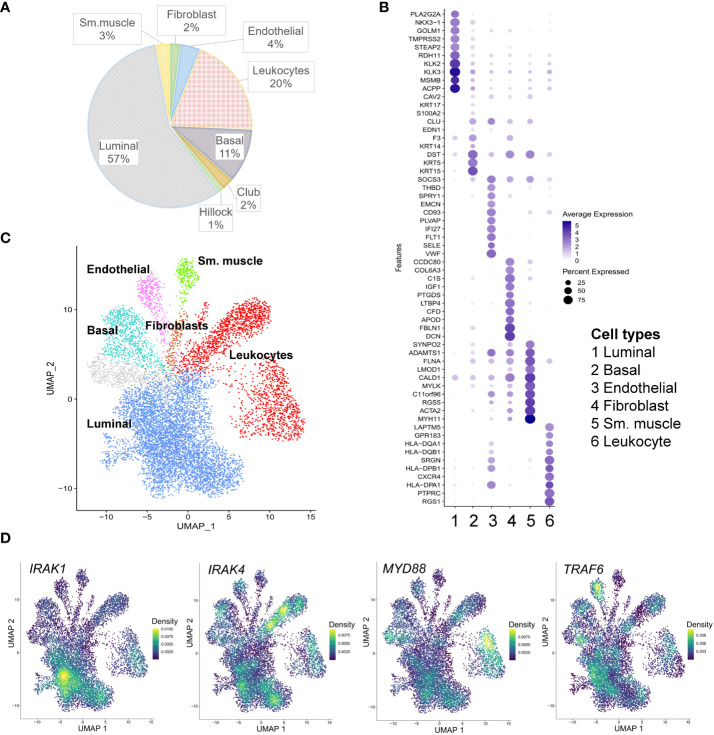
Single cell RNA-sequencing (scRNA-seq) analysis of *IRAK1*, *IRAK4*, *Myd88* and *TRAF6* expression in prostate cancer. **(A)** Cell type proportions within the merged scRNA-seq dataset from eight radical prostatectomy specimens (17). **(B)** Dot plot of top 10 marker genes for each of the displayed cell type clusters. Dot size and color represent the percentage of marker gene expression and average scaled expression value (minimum percentage expression 0.2%). **(C)** Cell type clusters detected in prostate cancer are presented in the uniform manifold approximation and projection (UMAP). **(D)** Density plots of *IRAK1, IRAK4, Myd88* and *TRAF6* expression in prostate cancer sm. muscle: smooth muscle cells.

The expression of genes encoding the main components of the Myddosome complex, *IRAK1*, *IRAK4* and *MYD88*, and the adaptor protein *TRAF6* were assigned to specific cell types within PCa using scRNA-seq data generated from eight radical prostatectomy (RP) specimens from men with localized PCa (17) (**Figure 1** and **Table S1**). Overall, 9643 cells underwent clustering analysis using principal component analysis (PCA) in the Seurat package (18). Using signature gene sets generated from single-cell profiling of normal prostate (20), PCa cells were classified as luminal (57%), basal (11%), hillock (1%), and club (2%) cells as the main epithelial cell lineage; endothelial (4%), fibroblast (2%), and smooth muscle (3%) cells as the main stromal lineage; and leukocytes (20%) as immune cells (**Figures 1A–C**; **Table S2**). The identities of the generated 16 cell clusters were assigned in compliance with the major (>80%) cell type represented in a cluster (**Figures 1B, C**). *IRAK1* is specifically enriched in luminal epithelial cell clusters. In comparison, *IRAK4* and *MYD88* were predominantly expressed in the leukocyte cluster and *TRAF6* was expressed in the endothelial cell cluster (**Figure 1D**).

### *IRAK1* overexpression in PCa is associated with differential methylation in *IRAK1*-DMR

**Figure 2:**
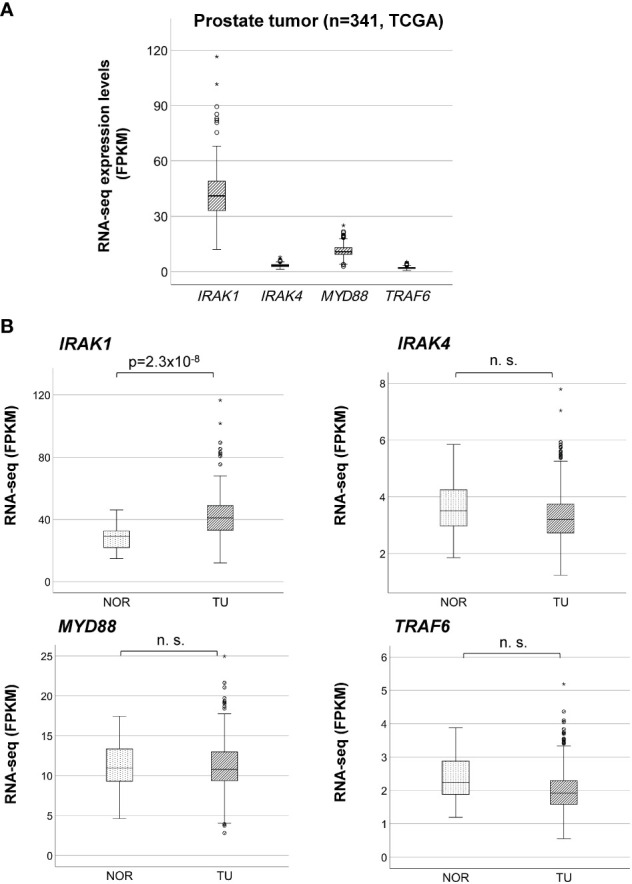
Overexpression of *IRAK1* in prostate cancer. **(A)** Comparison of bulk mRNA expression levels of *IRAK1*, *IRAK4*, *Myd88* and *TRAF6* in prostate cancer. Relative RNA expression is given in fragments per kilo base per million mapped reads (FPKM). **(B)** Differential mRNA expression analysis of *IRAK1*, *IRAK4*, *Myd88* and *TRAF6* in normal (NOR) and cancer (TU) prostate tissue. Significant difference was found only for *IRAK1* (eBayes, Benjamini-Hochberg adjusted p-value). n. s., not significant (p>0.05).

**Figure 3:**
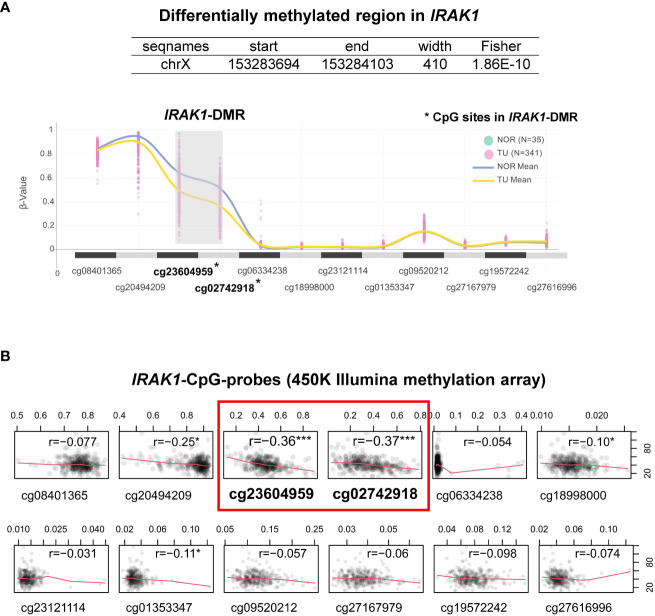
Characterization of the Differentially Methylated Region in *IRAK1* (*IRAK1*-DMR). **(A)** A snapshot of graphical user interface from ChAMP (Chip Analysis Methylation Pipeline) package showing a trend of DNA methylation levels (β-values) at different CpG-sites (presented as CpG-probes cg-number) in normal prostate (NOR) and prostate cancer tissue (TU). Lines represent mean methylation levels of analyzed CpG-probes (NOR, blue; TU, yellow). **(B)** Correlation plots of *IRAK1*-mRNA levels and *IRAK1*-methylation values in different CpG-probes (Spearman’s correlation coefficient r is indicated for each correlation analysis). *CpG-sites located within *IRAK1*-DMR. Significance levels: ***p<0.001, *p<0.05.

**Figure 4:**
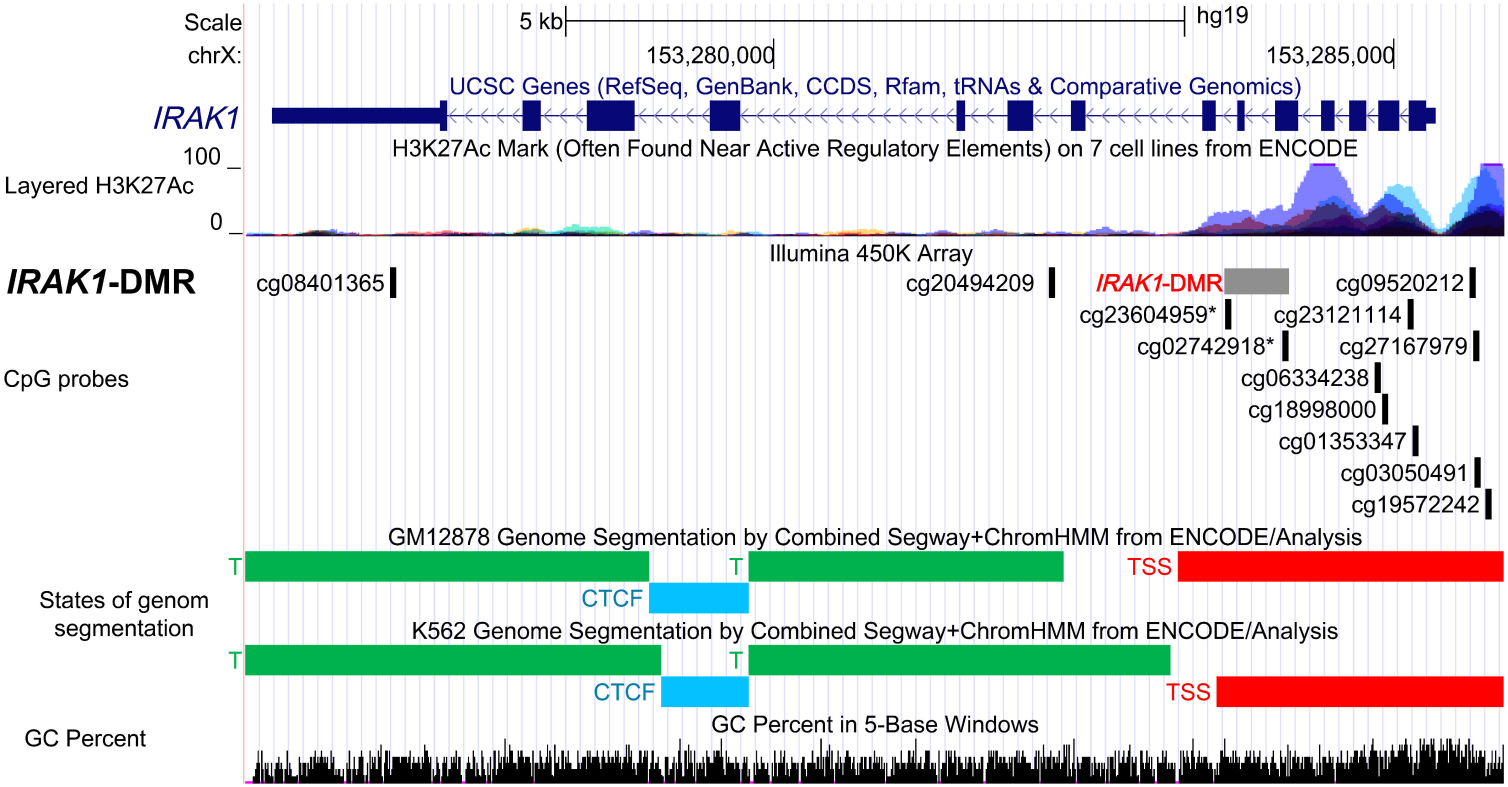
Functional characterization of the differentially methylated region in *IRAK1* gene (*IRAK1*-DMR) using ENCODE Data in the UCSC Genome Browser. Genomic position of *IRAK1-*DMR is shown in the USCS genome browser with reference to positions of CpG-probes (Illumina 450K Methylation array), enrichment of histone 3 lysine 27 acetylation (H3K27ac) peaks (layered H3K27ac peaks generated on seven human cell lines), predicted functional genomic states assembled by ChromHMM and Segway segmentation analysis in GM12878 and K562 cell lines (red, predicted promoter region including transcription start site, TSS). The GC percent track shows the densities of G (guanine) and C (cytosine).

By analyzing bulk RNA-seq data from TCGA using ebayes for differential expression, we found that *IRAK1* was significantly overexpressed in prostate tumor tissue (TU) compared to normal prostate tissue (TU, N=341; NOR, N=35; Benjamini-Hochberg adjusted p<2x10^-8^) (**Figures 2A, B**). Further differential analysis of *IRAK1* expression in non-CRPC and mCRPC specimens showed no difference between these two groups (non-CRPC, N=194; CRPC, N=99; Benjamini-Hochberg adjusted p>0.05) (data not shown). In contrast, *IRAK4*, *TRAF6* and *MYD88* were not differentially expressed in PCa and exhibited relatively low mRNA levels (**Figures 2A, B**). By analyzing 450K Illumina methylation array data from TCGA, we detected a PCa-associated differentially methylated region (DMR) within the predicted *IRAK1*-promoter (hg19 chrX:153283694-153284103) (TU, N=341; NOR, N=35; HMM-Fischer test, p<1.8x10^-10^, **Figure 3A**). Two CpG sites flanking this DMR were significantly hypomethylated in PCa (cg23604959 and cg02742918), and their methylation status showed an inverse correlation with *IRAK1* mRNA levels in PCa (n=341, Spearman coefficient =-0.36 and -0.37, respectively) (**Figure 3B**). The region including cg23604959 and cg02742918 (hg19 chrX:153283646-153284152) was determined as *IRAK1*-DMR in PCa (**Figures 3**, **4**).

### *IRAK1*-DMR is located in a regulatory active region

To assess the possible regulatory function of the *IRAK1*-DMR that was identified, we used data from ENCODE, which includes ChromHMM and Segway segmentation that predict and classify the functions of different gene regions (**Figure 4**). For the prediction of enhancer and promoter regions, Segway segmentation tools use features such as DNase-1 hypersensitivity and epigenetic marks. We found that *IRAK1-*DMR is located within the active promoter region and is enriched for histone 3 acetylated at lysine 27 (H3K27ac), a histone mark that is often found near active gene promoters (**Figure 4**).

**Figure 5:**
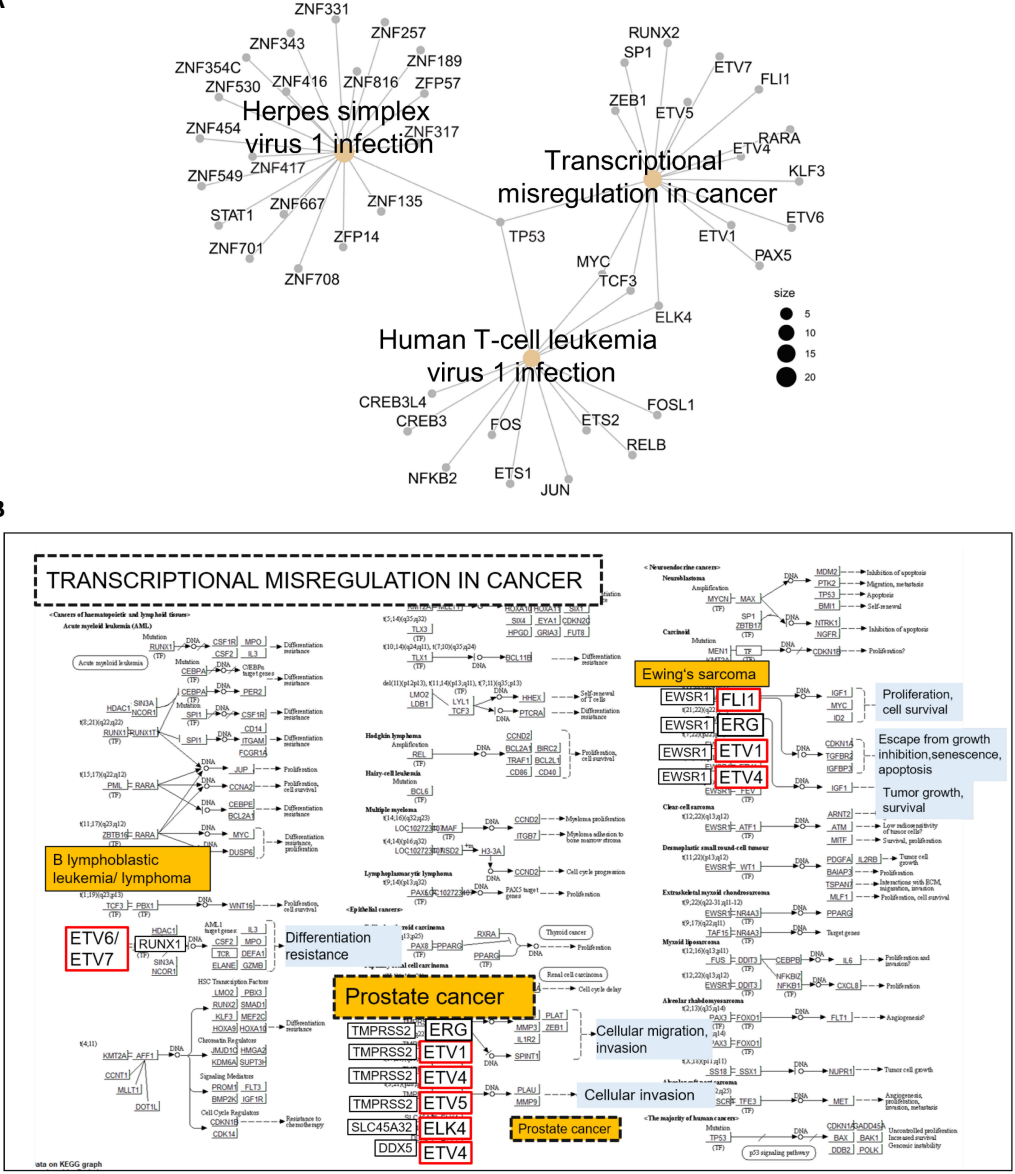
Kyoto Encyclopedia of Genes and Genomes (KEGG) pathway analysis of transcription factors (TFs) with predicted binding sites in *IRAK1*-DMR. TFs with binding scores ≥300 were considered (JASPAR-2022). **(A)** Cnet plot showing top three enriched KEGG pathways among TFs with predicted strong binding sites in *IRAK1*-DMR. **(B)** The enriched KEGG pathway ‘Transcriptional misregulation in cancer’ is shown together with implicated TFs exhibiting predicted binding sites in *IRAK1*-DMR (red framed).

Next, using the Transcription Factors Track database (JASPAR-2022), we identified 211 TFs with predicted strong binding sites in *IRAK1*-DMR (PWM relative score ≥ 0.8, p < 0.001) (**Table S4**). The biological function of TFs that bind specifically to *IRAK1*-DMR was further analyzed using KEGG enrichment analysis (**Figures 5A, B**). A substantial number of TFs were enriched in pathways associated with carcinogenic transformation and viral infection (**Figure 5A**). The top three enriched KEGG pathways were transcriptional dysregulation in cancer, herpes simplex virus 1 infection, and human T-cell leukemia virus 1 infection. Notably, 15 TFs were identified as members of the E26 transformation-specific (ETS) family of transcription factors, and genes encoding these TFs are known to fuse with hormone-regulated genes such as *TMPRSS2* (transmembrane protease serine 2), *SLC45A3* (solute carrier family 45 member 3, also known as prostate cancer-associated protein), and *DDX5 (*DEAD-Box Helicase 5) (24) (**Figure 5B**).

### Endogenous epigenetic status of *IRAK1*-DMR in PCa cells is critical for *IRAK1* activation upon treatment with epigenetic modifiers and bacterial infection

**Figure 6:**
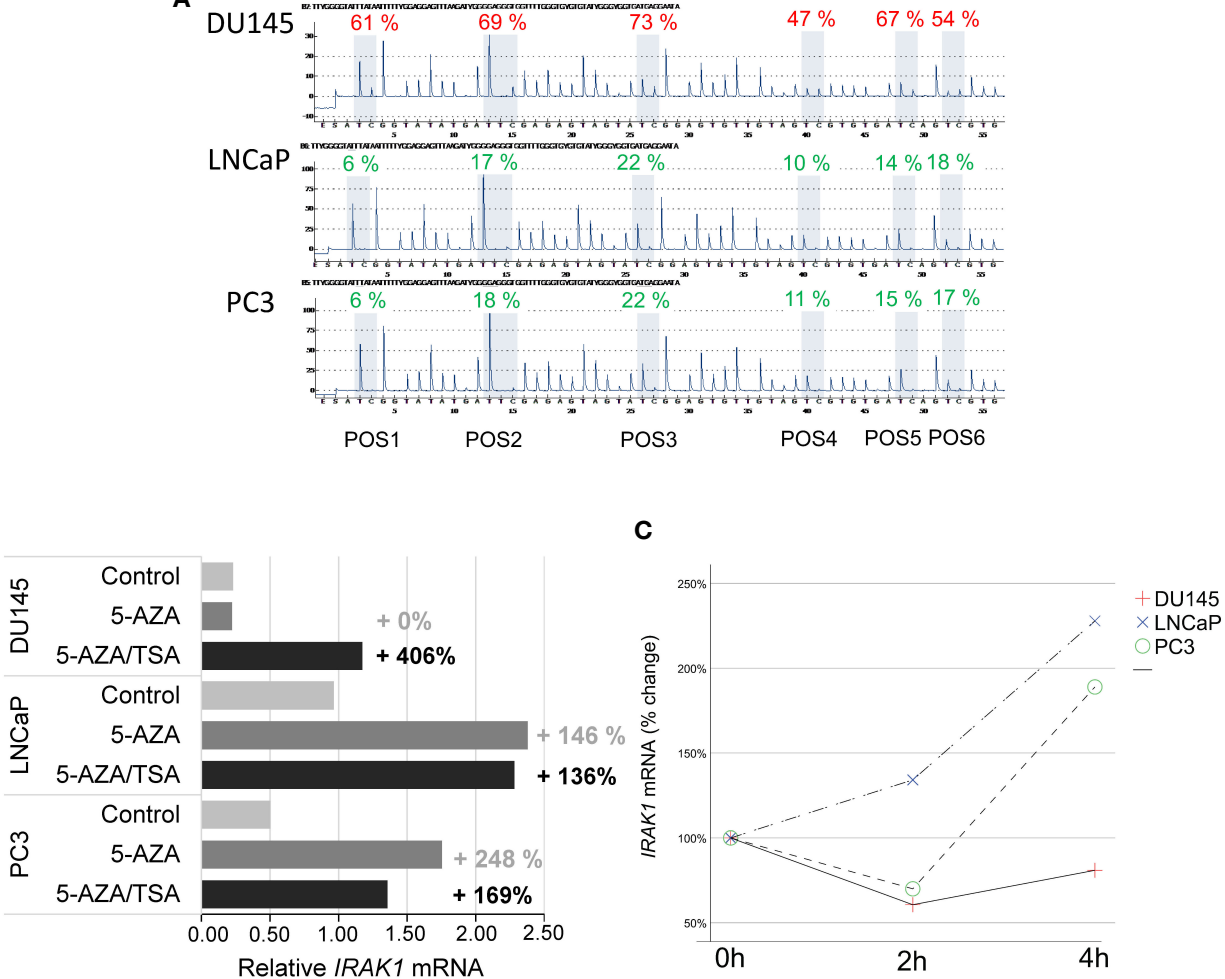
Analysis of DNA methylation in *IRAK1*-DMR and its impact on *IRAK1* expression using prostate cancer cell lines. **(A)** Pyrosequencing of *IRAK1*-DMR showed strong methylation in DU145 (47-69%) and weak methylation in LNCaP and PC3 cells (8-22%). **(B)** Effect of the DNA demethylating agent 5-Aza-2-deoxycytidine (5-AZA) and the histone-deacetylase inhibitor Trichostatin A (TSA) on *IRAK1* expression. In DU145, endogenous *IRAK1-*mRNA levels were low and increased over 400% after combined 5-AZA/TSA-treatment. LNCaP and PC3 cell lines showed higher endogenous *IRAK1* expression and lesser response to 5-AZA/TSA treatment than DU145. **(C)** Treatment with the uropathogenic *E*. *coli* strain UTI89 for 4 hours activated *IRAK1* expression in LNCaP and PC3, and had no effect in DU145 cells. Expression of *IRAK1* in untreated cells is set as 100%.

The decisive role of DNA methylation in *IRAK1-*DMR in *IRAK1* activation was analyzed and confirmed in cell culture experiments using bacterial infection. First, the methylation status of *IRAK1*-DMR was analyzed in DU145, LNCaP, and PC3 cell lines and was correlated with *IRAK1* expression (**Figure 6**). In DU145 cells, *IRAK1*-DMR hypermethylation (62% by pyrosequencing) was associated with markedly low endogenous gene expression (**Figures 6A, B**). A strong increase in *IRAK1* expression (>400%) was achieved by the combined treatment of DU145 cells with the DNMT1 inhibitor 5-AZA and HDAC inhibitor TSA (**Figure 6B**). 5-AZA treatment alone had no effect on *IRAK1* expression in DU145 cells (**Figure 6B**). In contrast, PC3 and LNCaP cells exhibited weak methylation in *IRAK1*-DMR (<14.5% by pyrosequencing) and showed >2-fold higher endogenous *IRAK1* expression than DU145 cells (**Figures 6A, B**). Treatment of PC3 and LNCaP cells with 5-AZA led to a moderate increase in *IRAK1* expression (146% and 248%, respectively) (**Figure 6B**). In PC3 and LNCaP, no further increase of *IRAK1* expression was achieved by combined treatment of cells with 5-AZA and TSA (**Figure 6B**).

Four hours after infection of PCa cell lines with uropathogenic *E. coli* UTI89 at a multiplicity of infection of 10, PC3 and LNCaP cells had a 2-fold increase of *IRAK1* expression. In contrast, in DU145 cells, which possess strongly hypermethylated *IRAK1*-DMR and low endogenous *IRAK1* expression, UTI89 infection did not induce *IRAK1* activation (**Figure 6C**).

## Discussion

In the scRNA-seq data analysis, *IRAK1* was mainly enriched in luminal prostate epithelial cells, representing the majority of cells detected in PCa. In contrast, in PCa, *IRAK4* and *MYD88* were predominantly expressed in leukocytes, and *TRAF6* was predominantly expressed in endothelial cells. This result implies a distinctive role for *IRAK1* in epithelial cells in PCa in a Myddosome-independent manner. Prior studies have demonstrated that IRAK1 is biologically active in a Myddosome-independent manner by interacting with alternative proteins and even independent of its catalytic function. Several substrates that interact with IRAK1 have been identified, including IRAK1 itself, Tollip, E3 ligase Pellino, transcription factor IRF7 (interferon regulatory factor 7), and intracellular adaptor protein TRIF (TIR-domain-containing adapter-inducing interferon-β (TRIF) (25–29). It has been shown that IRAK1 does not necessarily need catalytic activity to exert its function. Catalytically inactive IRAK1 with a point mutation at the catalytic site and a splice variant lacking part of the kinase domain were able to induce NF-kB activation (30, 31).

Consistent with the results of the scRNA-seq data analysis, bulk RNA sequencing data from TCGA showed in comparison to normal prostate a PCa-associated overexpression of *IRAK1.* No difference in *IRAK1* expression has been found between non-CRPC and mCRPC specimens. The remaining TLR signaling genes, *IRAK4*, *TRAF6* and *Myd88*, were not differentially expressed in PCa and exhibited relatively low mRNA levels. Overexpression of *IRAK1* has been found in several human carcinomas, including breast, endometrial, lung, and liver cancers, and is significantly associated with poor survival and unfavorable clinical parameters (32–36). Androgen deprivation therapy induces in PCa cells apoptosis and cell death. However, IRAK1 has been shown to have an antiapoptotic effect in cancer cells, and to contribute to developing resistance to radiotherapy as well as to paclitaxel and methotrexate treatment (14, 37, 38). It is known that androgens enhance the glycolytic metabolism and lactate export in PCa cells. Here, the conversion from pyruvate to lactate, which is catalyzed by lactate dehydrogenase (LDH) proteins including the androgen receptor target LDHA1 presents a critical step (39). Current evidence, gathered primarily from studies on beta-cells in pancreatic islets, suggests that LDH release is reduced in the absence of IRAK1 (40). In cell culture and xenograft tumor models, gain of IRAK1 promotes tumorigenic growth and metastasis of breast, head neck, and hepatocellular carcinoma. In accordance with that, specific inhibition of IRAK1 using endogenous inhibitors, e.g. mir-146a or shRNA, has an anti-tumorigenic effect on cells (34, 41, 42).

By analyzing 450K Illumina methylation array data generated in PCa, we discovered a differentially methylated region, *IRAK1*-DMR. The potential regulatory function of the identified *IRAK1*-DMR was assessed using ENCODE data, and supported by its localization within the active *IRAK1* promoter region and enrichment of the activating histone mark H3K27ac. It is well known that over 70% of regulatory DNA elements marked by H3K27ac are active and positively affect transcription *in vivo* (43). Importantly, the presence of H3K27ac and low DNA methylation preserves the accessibility of transcription factor-binding sites at the enhancer and promoter regions (44). In our study, TFs with strong binding affinity for *IRAK1*-DMR were found to represent key components of canonical pathways associated with carcinogenic transformation and virus infection, such as transcriptional misregulation in cancer, herpes simplex virus 1 infection, and human T-cell leukemia virus 1 infection. Notably, a considerable number of members of the ETS family of TFs were found among TFs with strong binding sites on *IRAK1*-DMR. In approximately 50% of PCa, ETS members are known to fuse with hormone-regulated genes such as *TMPRSS2*, prostate cancer-associated protein (*SLC45A3*), and RNA helicase *DDX5*, as reviewed by Nicholas and colleagues (24). Our results from integrative bioinformatic analyses provide convincing evidence for a decisive role of the DNA methylation status of *IRAK1*-DMR in transcriptional activation of *IRAK1*. These findings were further supported by our *in vitro* cell culture experiments, showing in the PCa cell line DU145 that weak endogenous *IRAK1*-mRNA expression is correlated with hypermethylation of *IRAK1*-DMR and can be dramatically increased (>400%) by combined treatment with the DNMT1 inhibitor 5-AZA and the HDAC inhibitor TSA. In contrast, weak DNA methylation in *IRAK1*-DMR, as was found in the PCa cell lines PC3 and LNCaP, was accompanied by a 2-3-fold higher endogenous *IRAK1*-mRNA expression than in DU145 cells, and with only a moderate activation of *IRAK1* expression upon 5-AZA and TSA treatment (maximum 169%). Importantly, different endogenous methylation statuses in *IRAK1*-DMR and expression levels of *IRAK1*, detected in DU145, LNCaP, and PC3 cells, respectively, were decisive determinants for the degree of *IRAK1* activation upon uropathogenic *E. coli* infection. LNCaP and PC3 cells were able to activate *IRAK1* 4 h after infection, but DU145 cells did not show any response to *IRAK1* expression.

The use of immune adjuvants is of growing importance in PCa therapy. Studies on *IRAK1* knockout mice and experiments with a specific endogenous *IRAK1* inhibitor, miR-146 indicate a clinical advantage for selective *IRAK1* inhibition  (45, 46). To date, only a few compounds, including the recently described chemical compound Jh-X-119-01 and the already established organic compound pacritinib (known as a potent tyrosine kinase inhibitor of JAK2/FLT3) are known to selectively inhibit IRAK1 (47, 48). Our data emphasize that determining the endogenous DNA methylation status of *IRAK1*-DMR and expression in PCa is of potential value in predicting the ability of PCa cells to activate IRAK1 and should be considered in setting up individualized PCa treatment algorithms in the clinic.

## Data availability statement

Publicly available datasets were analyzed in this study. The datasets presented in this study can be found in Gene Expression Omnibus (GEO) database: GSE176031. The accession numbers of used data can be found in the **Table S1**.

## Author contributions

TD and US designed the study. VB performed PCR assays. ML performed *E. coli* infection experiments. TD performed PCa cell culture experiments and bioinformatic data analysis. FW provided resources. TD and US wrote the paper. All authors contributed to the article and approved the submitted version.

## Funding

The study was supported by a Research Grant from the University Medical Center Giessen and Marburg to TD and FW (UKGM, project Nr. 6/2012 GI), and the German Research Foundation (DFG) to US and FW (GRK1871 “Molecular pathogenesis of male reproductive disorders”, project 5).

## Acknowledgments

We would like to thank Editage (www.editage.com) for English language editing.

## Conflict of interest

The authors declare that the research was conducted in the absence of any commercial or financial relationships that could be construed as a potential conflict of interest.

## Publisher’s note

All claims expressed in this article are solely those of the authors and do not necessarily represent those of their affiliated organizations, or those of the publisher, the editors and the reviewers. Any product that may be evaluated in this article, or claim that may be made by its manufacturer, is not guaranteed or endorsed by the publisher.
