# Supplementary material for: Interleukin-1 receptor associated kinase 1 (IRAK1) is epigenetically activated in luminal epithelial cells in prostate cancer
Source: Front Oncol. 2022 Sep 26;12:991368. doi: 10.3389/fonc.2022.991368 (PMC9549294; doi:10.3389/fonc.2022.991368)
Supplement: Supplementary file 1 [file DataSheet_1.docx]

**Supplementary Table S1** Eight radical prostatectomy patients and corresponding single-cell RNA-seq data sources used in our study (Song et al., 2022)

| **Patient: ID** | **GEO-Accession** | **Tissue specimen** | **PSA** | **GS** |
| --- | --- | --- | --- | --- |
| P4: PR5186 | GSM5353224 | Radical Prostatectomy Tumor | 7.6 | 4+5 |
| P5: PR5196 | GSM5353225  GSM5353226 | Radical Prostatectomy Tumor | 19.6 | 3+4 |
| P6: PR5199 | GSM5353227  GSM5353228  GSM5353229 | Radical Prostatectomy Tumor | 16.5 | 4+3 |
| P7: PR5269 | GSM5353232  GSM5353233 | Radical Prostatectomy Tumor | 4.1 | 3+4 |
| P8: PR5249 | GSM5353236  GSM5353237 | Radical Prostatectomy Tumor | 5.2 | 3+4 |
| P9: PR5251 | GSM5353240 | Radical Prostatectomy Tumor | 7.7 | 3+4 |
| P10: PR5254 | GSM5353243  GSM5353244 | Radical Prostatectomy Tumor | 7.8 | 4+3 |
| P11: PR5261 | GSM5353245  GSM5353246  GSM5353247 | Radical Prostatectomy Tumor | 3.89 | 3+4 |

**Abbreviations:** ID: identity; PSA: prostate specific antigen (ng/ml); GS: Gleason score

**Supplementary Table S2** Cell type marker defined for normal prostate (Henry et al., 2018) and used in our study for cell clustering and cell type identification

| **Cell type** | **Marker** |
| --- | --- |
| **Luminal epithelial cells** | ABHD2, ACPP, ALDH1A3, CIB1, DBI, DHRS7, DSC2, ELOVL5, FDPS, FLJ20021, FXYD3, GOLM1, H2AFJ, HMGCS1, IL6ST, INSIG1, JUN, KLK2, KLK3, KLK4, LINC00844, LMO7, MSMB, MT1F, MT1G, NKX3-1, PART1, PLA2G2A, PLPP1, PMEPA1, PPP3CA, RAB27B, RASEF, RDH11, RPS27L, SERF2, SLC12A2, SLC39A6, SLC44A4, SMIM14, SORD, STEAP2, TMC5, TMPRSS2, TSPAN8, UQCRQ |
| **Basal epithelial cells** | CAV1, CAV2, CLU, DEFB1, DST, EDN1, F3, FHL2, HBEGF, HSPA1B, KRT14, KRT15, KRT17, KRT5, PHLDA1, RGCC, S100A2, STK17A, ZFP36L2 |
| **Club epithelial cells** | ANKRD36C, BIK, CEACAM5, CEACAM6, CP, CRABP2, CXCL6, CYP1B1, CYP3A5, FAM3D, FCGBP, FOXO3, FTH1, GLUL, LCN2, LINC01207, MMP7, PDZK1IP1, PI3, PIGR, PPDPF, REEP3, RHOV, S100A6, SAA1, SCCPDH, SCGB3A1, SLPI, SRD5A3, TMEM165, TMEM45B, TSPAN3, VSIG2, WFDC2, |
| **Hillock epithelial cells** | AKR1C2, APOBEC3A, AQP3, C9orf16, CCL20, CDKN2B, CLCA4, CLDN4, CLDN7, CSTB, DAPP1, DDIT4, DHRS9, EMP1, HEBP2, IGFBP3, IL1RN, KRT13, LYPD3, MUC4, OAS1, PIM1, PPARG, SDC1, SDCBP2, SERPINB1, SNCG, TMPRSS4, TRIM31 |
| **Endothelial cells** | ACKR1, ADAMTS9, ADGRL4, ANGPT2, AP1S2, APP, AQP1, ARPC1B, ARPC5L, ASPH, ATP1A1, C4orf32, CALCRL, CARHSP1, CD200, CD59, CD93, CDC37, CDC42EP3, CFLAR, CLDN5, CNKSR3, CRIP2, CSF3, CXCL2, DUSP23, DUSP6, ECSCR.1, EGFL7, EMCN, ENG, ESAM, ETS2, FKBP1A, FLT1, FXYD5, GJA1, GNG11, HLA-B, HLA-E, HMOX1, HSPG2, ICAM1, IFI27, IL3RA, INSR, ITM2A, LCN6, LMO2, LUZP1, MCTP1, MGST2, MMP1, MRPL33, MSN, MYL12A, NOP10, NQO1, PCAT19, PDLIM1, PECAM1, PGF, PLVAP, PNP, PPFIBP1, PRSS23, RAB13, RAI14, RAMP2, RAMP3, RCAN1, RDX, RHOC, RNASE1, RND1, S100A10, SELE, SH3BGRL3, SLC7A11, SLC9A3R2, SOCS3, SOX17, SPRY1, SQSTM1, STC1, SWAP70, TCF4, THBD, TM4SF1, TM4SF18, TMEM255B, TMSB10, TPM3, TSPAN7, TXN, TXNRD1, UBE2J1, UPP1, VWF, WWTR1, ZEB1, ZNF385D, |
| **Fibroblast cells** | ADM, AEBP1, AKAP12, ANXA5, APOD, APOE, ARSG, BNIP3L, BTG3, C1R, C1S, C7, CCDC80, CCK, CCND1, CEBPB, CFD, CFH, CLMP, COL6A1, COL6A2, COL6A3, CREB5, CST3, CTSK, CXCL1, CXCL8, DCN, DKK3, DNAJB9, DPT, EFEMP1, EMP3, FBLN1, FBLN2, FGF2, FGF7, FGFR1, FST, FSTL1, G0S2, GEM, GPM6B, GSN, GSTM3, GSTO1, HERPUD1, HSD11B1, HSPA5, IER3, IGF1, IGFBP2, IL32, IL33, IL6, INHBA, KCNK1, LAPTM4A, LGALS3BP, LIF, LMO4, LPAR1, LTBP4, LUM, MATN2, MFAP4, MGP, MMP2, MPZL1, NBL1, OSBPL8, PCOLCE, PCOLCE2, PDGFRA, PDPN, PID1, PLAT, PLIN2, PLPP3, PRELP, PRRX1, PTGDS, PTGS2, PTN, RARRES2, REEP5, RHOBTB3, RHOQ, RND3, SDC2, SERPINE2, SERPING1, SFRP1, SFRP2, SLC39A14, SNCA, SPOCK3, SPON2, TFPI, TFPI2, THAP2, THBS1, THBS2, TIMP1, TNFAIP6, TNFRSF12A, TWIST2, UAP1, UFM1 |
| **Neuroendokrine cells** | ASCL1, BEX1, C3orf14, C4orf48, CALCA, CGA, CHGA, CHGB, DNAJC12, DPYSL3, FAM102A, FGF14, GRP, HES6, IFI6, IZUMO4, KIF1A, KLK12, MAOB, MAP9, MIAT, MS4A8, NAAA, PCSK1N, PHGR1, PKIB, QDPR, QPCT, SCG2, SCG5, SCGN, SLC38A11, SLITRK6, SPOCK1, ST18, SYP, SYT1, TAGLN3, TFF3, TMEM176A, TMEM176B, TPH1, TUBB2B, UCHL1, ZACN |
| **Smooth muscle**  **cells** | ACTA2, ACTG2, ADAMTS1, ADAMTS4, ADRA2A, APOLD1, ATF3, BCAM, BGN, C11orf96, C2orf40, CALD1, CCDC107, CKB, CNN1, CPM, CREM, CRISPLD2, CSRP1, DES, DSTN, EFHD1, EPAS1, EPS8, FHL1, FLNA, FOXC2, FRZB, FXYD1, GJA4, GPX3, HES4, HSPB1, ID4, ISYNA1, JUNB, KCNE4, KIAA0040, KLF9, LBH, LGI4, LMOD1, LPP, MAT2A, MFGE8, MT1A, MT1M, MT1X, MTHFD2, MYH11, MYL6, MYLK, NDUFA4, NDUFS2, NOTCH3, NR4A1, PCP4, PDGFRB, PDK4, PDLIM3, PLN, PPP1R14A, PRKCDBP, PTP4A3, RCAN2, RGS16, RGS5, RHOB, RRAD, S100A4, SH3BGRL, SLC25A4, SMTN, SORBS2, STEAP4, SYNM, SYNPO2, TCEAL4, TINAGL1, TPM1, TPM2, ZFHX3, ZNF331 |
| **Leukocytes** | ACSL1, AIF1, ALOX5AP, ANXA1, ARHGDIB, ARL4C, ARPC3, ARRB2, ASAH1, BCL2A1, C1orf162, C1QA, C1QB, C1QC, C5AR1, CACYBP, CAPG, CCL3, CCL3L3, CCL4, CCL4L2, CCR7, CD163, CD37, CD48, CD53, CD68, CD83, CHCHD10, CMTM6, CORO1A, COTL1, CTSB, CTSD, CTSH, CTSS, CTSZ, CXCL16, CXCR4, CYBA, CYTIP, DNAJA4, DNAJB1, DNAJB6, DOK2, DUSP2, EREG, EVI2A, EVI2B, EZR, FABP5, FAM26F, FAM49B, FCER1G, FCGR2A, FGL2, FYB, GPR183, GPSM3, GPX1, GRN, HCLS1, HCST, HLA-DMA, HLA-DMB, HLA-DPA1, HLA-DPB1, HLA-DQA1, HLA-DQB1, HSP90AA1, HSPA6, HSPD1, HSPE1, HSPH1, ID2, IER5, IL1B, IL1R2, ITGAX, ITGB2, KLF6, KYNU, LAPTM5, LCP1, LIPA, LSP1, LST1, LYZ, MAFB, MS4A4A, MS4A6A, MS4A7, MXD1, NINJ1, NPC2, OGFRL1, OLR1, PDE4DIP, PLEK, PSAP, PTPRC, REL, RGS1, RGS10, RGS2, RIPK2, RNASE6, RNASET2, RNF130, SAMSN1, SERPINB9, SMAP2, SPI1, SRGN, TAOK3, TSC22D3, TYMP, TYROBP, VAMP8, VSIG4, ZFAND2A |

**Supplementary Table S3** Primer sets used for pyrosequencing and RT-qPCR

| **Assay** | **Primer** | **Sequence 5’-3’** | | **PCR**  **Product** | |
| --- | --- | --- | --- | --- | --- |
| Pyro-  sequencing | Sequence to analyze* | 5’-TYGGGGTATTTATAATTTTTYGGAGG  AGTTTAAGATYGGGGAGGGTGGTTTTGGGTGYGTGTATYGGGYGGTGATGAGGAATA-3’ |  | |  |
|  | BS_IRAK1_FW | GAGTTTAGTGTTTTTTTTGTAGGGAGTT | 176bp | |  |
|  | BS_IRAK1_REV | CCTTCAACCTCTTCACAACATAC |  | |  |
|  | BS_IRAK1_seq | TGGTTTTTTTGTGAGATTT |  | |  |
| RT-qPCR | GAPDH-FW | TGGAGAAGGCTGGGGCTCAT | 176bp | |  |
|  | GAPDH-REV | GACCTTGGCCAGGGGTGCTA |  | |  |
|  | IRAK1-FW | CCAAGTATCTGGTGTACGAGAGG | 153bp | |  |
|  | IRAK1-REV | ATGGAGCAGCCCCACTGT |  | |  |
| **Abbreviations:** RT-qPCR: reverse transcription followed by quantitative polymerase chain reaction; IRAK1: Interleukin-1 receptor-associated kinase 1; GAPDH: glycerinaldehyd-3-phosphat-Dehydrogenase; FW: forward primer; REV: reverse primer; * sequence after bisulfite (BS) treatment (Y: C+T sites); bp: base pairs | | | | |  |

**Supplementary Table S4** Functional annotation of TFs with predicted strong binding sites in *IRAK1*-DMR using Kyoto Encyclopedia of Genes and Genomes pathway analysis

| **Description** | **Adj. p-value*** | **TFs binding in *IRAK1*-DMR** |
| --- | --- | --- |
| Transcriptional misregulation in cancer | 2.05E-08 | **ELK4, ETV1, ETV4, ETV5, ETV6, ETV7, FLI1, KLF3, MYC, PAX5, RARA, RUNX2, SP1, TCF3, TP53, ZEB1** |
| Herpes simplex virus 1 infection | 1.20E-05 | **STAT1, TP53, ZFP14, ZFP57, ZNF135, ZNF189, ZNF257, ZNF317, ZNF331, ZNF343, ZNF354C, ZNF416, ZNF417, ZNF454, ZNF530, ZNF549, ZNF667, ZNF701, ZNF708, ZNF816** |
| Human T-cell leukemia virus 1 infection | 2.64E-05 | **CREB3, CREB3L4, ELK4, ETS1, ETS2, FOS, FOSL1, JUN, MYC, NFKB2, RELB, TCF3, TP53** |
| Parathyroid hormone synthesis, secretion and action | 5.97E-05 | **CREB3, CREB3L4, FOS, GCM1, GCM2, JUND, NR4A2, RUNX2, SP1** |
| Osteoclast differentiation | 2.29E-04 | **FOS, FOSB, FOSL1, FOSL2, JUN, JUND, NFKB2, RELB, STAT1** |
| Chemical carcinogenesis - receptor activation | 1.91E-03 | **ARNT, CREB3, CREB3L4, ESR2, FOS, JUN, KLF4, KLF5, MYC, PPARA** |
| Breast cancer | 3.32E-03 | **ESR2, FOS, JUN, MYC, NFKB2, SP1, TCF7, TP53** |
| Th1 and Th2 cell differentiation | 7.59E-03 | **FOS, JUN, RBPJ, RUNX3, STAT1, TBX21** |
| Prostate cancer | 8.96E-03 | **CREB3, CREB3L4, ETV5, TCF7, TP53, ZEB1** |
| Estrogen signaling pathway | 9.26E-03 | **CREB3, CREB3L4, ESR2, FOS, JUN, RARA, SP1** |
| Signaling pathways regulating pluripotency of stem cells | 9.64E-03 | **ESRRB, KLF4, MYC, MYF5, TBX3, TCF3, TCF7** |
| Amphetamine addiction | 9.64E-03 | **CREB3, CREB3L4, FOS, FOSB, JUN** |

**Abbreviations:** *IRAK1*-DMR: differentially methylated region in prostate cancer localized in predicted *IRAK1* promoter; * Benjamini-Hochberg adjusted p-value; TFs: transcription factors
